# Induced pluripotent stem cells in the treatment of ischemic stroke

**DOI:** 10.1515/jtim-2024-0043

**Published:** 2025-07-30

**Authors:** Qian Zhang, Xintong Song, Yi Ju, Xingquan Zhao

**Affiliations:** Department of Neurology, Beijing Tiantan Hospital, Capital Medical University, Beijing, China; China National Clinical Research Center for Neurological Diseases, Beijing Tiantan Hospital, Capital Medical University, Beijing, China; Research Unit of Artificial Intelligence in Cerebrovascular Disease, Chinese Academy of Medical Sciences, Beijing, China; Center of Stroke, Beijing Institute of Brain Disorders, Capital Medical University, Beijing, China

## Introduction

Ischemic stroke has been a leading cause of death and disability worldwide, and brought a heavy economic burden, with an increasing trend for onset at a younger age. Stem cells transplantation has become a novel possible option for the treatment of ischemic stroke. Stem cells are a class of cells with the potential for self-renewal and differentiation into different cell types. Based on their source, differentiation potential, and characteristics, stem cells can be categorized into various types: (1) totipotential stem cells, which have the differentiation potential to form a complete individual, such as fertilized ovum;(2) subtotipotent stem cells, which have the potential to differentiate into a variety of cell tissues except for a complete individual, such as embryonic stem cells (ESCs) and induced pluripotent stem cells (iPSCs); (3) pluripotent stem cells, which have the potential to differentiate into specific cell lineage types, such as mesenchymal stem cells (MSCs), neural stem cells (NSCs), *et al*; and (4) unipotent stem cells, which can only differentiate into one cell type, such as endothelial progenitor cells (EPCs). Stem cells have been proved to have some therapeutic effects, including cell migration, neuroregeneration, neuroprotection, trophic support, immune modulation, angiogenesis and vasculogenesis, and neural circuit reconstruction.^[1]^ In general, differentiation potential and paracrine effects are the two main mechanisms of stem cells therapy. A number of clinical trials have been conducted which have thoroughly supported a strong safety profile for stem cell therapy in patients and have generated variable efficacy.

Pluripotent stem cells (PSCs) are a category of biological cells with unique potential for unlimited proliferation and differentiation into cells of all three germ layers. PSCs primarily exist as two main types: ESCs and induced iPSCs. iPSCs, which are generated from somatic cells by reprogramming using specific transcription factors into PSCs, can differentiate into all adult cell types, similar with ESCs. Despite iPSC-derived cell implantation in stroke patients might improve their recovery, the exact mechanisms remain not clear. Recent studies have revealed some potential mechanisms, including neuroprotective effects, inflammation-suppression, restore of cerebral blood flow, reduction in cerebral edema, and reduction in autophagy.^[2,3]^ Compared to ESCs/MSCs/NSCs, iPSCs have some advantages, such as broad applications with the ability to differentiate into any type of cells including neural cells, neural precursor cells(NPCs), and vascular endothelial cells and strong *in vitro* expansion capability, great source for disease modeling, ideal starting materials for cell and gene therapies, can be derived from various kinds of cells, as well as easier methods are available for cell isolation than using bone marrow or embryonic stem cells, with no ethical problems compared with ESCs. iPSCs also have some disadvantages, such as lack of clinical trial evidence, and reprogramming may lead to epigenetic or genetic alterations.^[2]^ iPSCs provide opportunities for research on stem cells. Nowadays, some iPSC-derived cellular products have been used in the undergoing clinical trials.^[4]^

## IPSC-derived NSCS

NSCs possessing multipotent and self-renewing capabilities exist in the intact brain, particularly in the hippocampal dentate gyrus (SGZ) and lateral ventricles (SVZ). Following a stroke, a limited endogenous regenerative process will begin and NSCs will migrate to the infarcted area. An increasing amount of evidence has indicated that NSCs derived from iPSCs, possess potential for therapeutic application. iPSC-derived NSCs can improve early functional recovery by differentiating into neurons and oligodendrocytes and migrating to the ischemic region.^[5]^ Moreover, an animal study has demonstrated that iPSC-NSCs reduced expression of proinflammatory factors, microglial activation, adhesion molecules, and reduced damage of blood brain barrier (BBB).^[6]^ NSCs used in clinical trials were administrated by stereotaxic intracerebral injection, not restricted by the BBB. However, NSCs are usually used in chronic, stable patients at late stages after ischemic stroke, with a limitation of invasiveness. iPSC-derived human forebrain neural progenitor cells injection (hNPC01) is being used in patients with chronic ischemic stroke (Phase I, NCT06299033) to explore the safety and tolerability.

## IPSCS-derived MSCS

MSCs are non-hematopoietic stem cells that possess high proliferation, self-renewal, and multipotent differentiation properties, and exhibit the potential in the repair of neuron injuries. MSCs, endowed with regenerative capabilities and a paracrine mechanism, exert neurotherapeutic effects through secretion of neurotrophic factors, delivery of exosomes, modulation of inflammation, reduction of oxidative stress, and enhancement of synaptic plasticity.^[4]^ iPSC-derived MSCs have been used as an alternative resource of tissue-derived MSCs for unlimited expansion and biomanufacturing. Previous studies showed MSCs induced from iPSCs derived from different sources, such as dental pulp, periodontal ligament, and lung tissue, exhibited similar characteristics in self-renewal, multilineage potential, and surface marker expression.^[4]^ Compared with the donor matched UC-MSC, iPSC-derived MSCs exhibited higher neural differentiation potential and showed higher immunosuppressive function *in vitro*.^[4]^ In preclinical experiments, iPSC-derived MSCs exhibited the capacity to inhibit the activation of glial cells in the cerebral ischemia-reperfusion injured region^[7]^ and restore the metabolic activity of neurons through mitochondrial respiration.^[8]^ Although research *in vitro* has shown MSCs’ multilineage differentiation capacity, as well as their immunomodulatory and anti-inflammatory effects through paracrine action, their behavior in the human body is not yet fully clear,^[4]^ especially for their differentiation capacity.

## IPSC-derived EPCS

EPCs constitute a pool of circulating bone-marrow derived cells that mobilize after an ischemic injury with the potential to incorporate into the damaged endothelium, to form new vessels, or to secrete trophic factors stimulating vessel remodeling.^[9]^ The circulating levels of EPCs are altered after stroke, and EPCs have been proved to promote neurorepair in preclinical models of cerebral ischemia. EPCs contribute to tissue repair by both direct incorporation into remodeling vessels or by indirect secretion of proteins or molecule-containing vesicles supporting cell communication, as cellular mediators of vascular remodeling and repair in the poststroke brain during recovery.^[9]^ The angiogenic ability of EPCs is probably the most distinguishable characteristic over other stem cells.^[10]^ However, whether EPC-based therapy is the safest and has the greatest efficacy over other types of stem/progenitor cells is still unknown. The allogeneic EPCs derived from iPSCs have the advantages of short preparation time, unrestricted yield and high product consistency. Human iPSC-derived endothelial cells can promote CNS remyelination *via* brain-derived neurotrophic factor (BDNF) and mTORC1 pathway.^[11]^ The ongoing Phase I clinical trial used intravenously allogeneic iPSC-derived EPCs (“ALF201” injectable solution) in patients with acute ischemic stroke (NCT05993884) and will bring a new perspective in cell-based therapy.

## IPSC-derived exosomes

One of the main mechanisms of stem cells is by paracrine, and extracellular vesicles (EVs) play an essential role in this process. Exosomes perform a crucial role as paracrine factors of stem cells in cell-to-cell communication and in promoting the microenvironment. Their impacts include repairing after stress, removing toxic agents, and increasing tolerance to oxidative stress. Exosomes can cross the BBB and have less immunogenicity, compared to stem cells.^[2]^ Exosomes derived from stem cells have shown therapeutic benefits in various diseases, such as acute myocardial ischemia- reperfusion. However, the mechanism of iPSC-exosomes in ischemic stroke is still uncertain. Phase I clinical trial is evaluating safety and preliminary efficacy of intravenous iPSC-derived exosomes l (GD-iExo-003) in acute ischemic stroke (NCT06138210).

## Difficulties and challenges

Tumorigenicity, heterogeneity, immune rejection, cell survival, and migration are some concerns associated with stem cell therapy. Due to the potential of tumor formation being a significant concern in cell implantation, it is essential to develop stable and highly sensitive methods for detecting residual iPSCs and unintended differentiated cell remnants to minimize the risk of tumorigenicity. It is required to deal with product heterogeneity in the development of iPSC products, and it can be further analyzed through single-cell transcriptome sequencing method to evaluate different subpopulations. Additionally, iPSCs holds the potential to reducing the risk of immune rejection during cell therapy as autografts, or by screening human leukocyte antigen (HLA) monotypic homozygous populations as donors. Furthermore, more studies are required to compare iPSC derived and primary tissue derived cellular products in terms of regeneration, differentiation, and immunomodulatory capabilities.^[4]^

Consistent quality and efficacy of iPSC-derived cellular products from different sources or different differentiation pathway are necessary to be ensured by establishing standardized quality control procedures and manufacturing standards, to transit to clinical application with better consistency, reliability, and safety.

## Perspective

iPSC-derived cellular products might potentially become a prospective component of ischemic stroke treatment in the future.
